# Supplementary material for: Are carbon emissions trading and green financial instruments synergistic? -Comprehensive quantitative research based on content analysis
Source: PLoS One. 2024 Mar 7;19(3):e0298601. doi: 10.1371/journal.pone.0298601 (PMC10919601; doi:10.1371/journal.pone.0298601)
Supplement: S1 Data — (ZIP) [file pone.0298601.s001.zip › Data/地级市Z_F工作报告.zip!/║╙▒▒/╠╞╔╜╩╨/└┤╫╘╣1⁄2╓┌║┼í╛┬φ┐╦╩2╛▌═°í┐_2018.docx]

2017年工作回顾

2017年，是我们党和国家历史上具有特殊重要意义的一年。一年来，作为倍受习近平总书记亲切关怀的英雄城市，我们以迎接和学习宣传贯彻党的十九大为主线，坚定不移朝着总书记亲自为唐山描绘的“三个努力建成”宏伟蓝图阔步前进，谱写了习近平新时代中国特色社会主义思想在唐山大地的生动实践。

2017年，也是市十五届人民政府履职尽责的第一年。一年来，在省委、省政府和市委的坚强领导下，全市上下稳中求进、接续奋斗，全力以赴转方式、调结构、推进供给侧结构性改革，打赢了去产能、稳增长、治污染、惠民生等多场硬仗，开创了经济社会发展新局面。

一是突出项目支撑促投资稳增长，经济发展在转型升级中提速突破。以季度为单元组织项目集中开工和观摩测评，特别是加大新增规模以上工业企业考核权重，进一步强化了抓实抓成项目的实干导向，全市新开工亿元以上项目1007个、完成投资2554亿元，分别增长45.7%和22.2%;新增规模以上工业企业350家，数量位居全省首位。京唐城际、唐曹铁路、唐廊高速等基础设施完成投资1341亿元，迁曹高速一期建成通车。全市地区生产总值增长6.5%，规模以上工业增加值增长4.7%，一般公共预算收入增长7.1%，固定资产投资增长6.6%，实际利用外资增长8.3%，经济发展实现了稳中有升、稳中提质、进中突破。

二是聚焦供给侧结构性改革优增量调存量，质量效益在动能转换中不断提升。全年化解炼钢产能993万吨、炼铁产能576万吨，切实担当了全省去产能的“唐山责任”。工业投资和技改投资分别增长12.1%、12.7%，钢铁高附加值产品比重提高到21.5%。全市新增科技型中小企业1863家、高新技术企业80家，高铁装备、特种机器人成为京津冀最大的产业基地，高新技术产业增加值增长17.2%。装备制造业增加值增长16.3%，规模以上工业企业利润增长70.7%，新增注册企业数量增长21.5%，实体经济焕发出新的生机活力。农业供给侧结构性改革深入推进，农业产业化经营率提高到69%。

三是释放比较优势抓协同促开放，沿海经济在产业聚集中加速崛起。沿海板块实施亿元以上项目655个，完成投资934亿元。京冀曹妃甸、津冀芦汉两大协同发展示范区增势强劲，中林木材加工园等一批重大产业项目落地开工。曹妃甸国家级石化产业基地规划环评获批，产业项目聚集突破。唐山港整车进口口岸开放运营，内陆港达到10家，全年货物吞吐量5.7亿吨、集装箱吞吐量253万标箱，分别增长10.1%和30.7%。唐山机场旅客吞吐量突破50万人次、增长115.4%，货邮行吞吐量3876吨、增长82%。构建开放型经济新体制试点试验通过国家终期评估，唐钢塞尔维亚钢厂成为我国与中东欧国际产能合作的样板工程。

四是坚持“三城齐抓”增内涵补短板，城乡建设在协调并进中统筹发展。以优质板块拓展产业空间，京唐智慧港、站西片区、南湖CBD规划建设顺利推进，城南经济开发区获批省级开发区。建成中国铁路源头博物馆、中国(唐山)工业博物馆、中国(唐山)陶瓷博物馆等一批城市工业游核心项目，成功举办国际职业教育大会、中国工业旅游产业发展联合大会、唐山国际体博会、第二届中拉沙滩足球锦标赛等赛事展会活动，游客总数、旅游收入分别增长25%和32%，会展业直接收入增长26%，餐饮住宿业营业收入增长13.2%，城市人气活力不断增强。商贸、金融、电子商务等业态蓬勃发展，服务业增加值增长11%。深入开展县城建设攻坚和美丽乡村建设，常住人口城镇化率达到61.64%，122个村获评全国首批绿色村庄。全国文明城市创建实现“三连冠”。

五是强化问题导向破瓶颈治堵点，发展活力在改革创新中持续迸发。深化“放管服”改革，取消调整下放市级权力事项81项，新市民服务中心集中划转、进驻336项审批和服务事项，当场审批办结率提高3倍。市县两级行政审批局实现全覆盖。唐山重装集团与金隅冀东开启战略重组，新城建集团组建运行。扎实开展国家小微企业创业创新基地城市示范，引进京津科技成果和产业化项目49项，省级以上众创空间达到13家。创新化解工业用地遗留问题，盘活工业用地5213亩，305家停产半停产企业实现“腾笼换鸟”。新增主板上市企业2家、“新三板”挂牌企业10家。

六是回应群众关切办实事解难题，民生福祉在普惠共享中显著改善。全市居民人均可支配收入增长8.8%，城镇登记失业率控制在2.83%。棚户区改造1.5万套，204个省挂账房地产遗留问题基本清零，11.5万户居民办理不动产证。西南交大唐山研究生院挂牌招生。新妇幼医院开诊运营，市中心医院部分开诊。基本医疗保险实现跨省就医直接结算。新体育中心开工建设，青少年宫、科技馆达到开放条件，新工人文化宫向社会开放。唐山北立交等8个收费站撤站畅行。4547户贫困人口建档立卡，城乡低保标准全省领先。超额完成大气“国十条”PM2.5浓度比2013年下降33%的目标，“气代煤”改造6.7万户，森林覆盖率达到37.2%。完成潘大水库、邱庄水库网箱养殖清理，陡河水库围挡封闭，从根本上解决了多年形成的水污染隐患问题。成功创建国家食品安全城市，安全生产形势总体平稳。

七是履职尽责转作风抓落实，政府执行力在实干担当中不断提高。树牢“四个意识”，坚持依法行政，认真执行市人大及其常委会决议决定，自觉接受各方面监督，办理人大代表建议339件、政协委员提案536件，按时办复率100%。严格执行中央八项规定，驰而不息整治“四风”，全市“三公”经费下降16.2%。健全完善市政府法律顾问和人大代表、政协委员、群众代表参与决策机制，政府决策科学化、法治化水平有效提升。持续开展“爱唐山、做贡献”主题实践活动和市直部门工作交流会，塑树以业绩贡献为实干导向的激励约束机制，推动形成了政府系统担当尽责、干事创业的正气新风。 这一年，我们坚决贯彻落实新发展理念，有效应对去产能、调结构、治污染等多重压力挑战，巩固提升来之不易的向好局面，全市发展“稳”的基础更加坚实，“进”的态势更加强劲，唐山步入了加速转型升级、迈向高质量发展的良性轨道。这一年，我们接续释放后世园效应，干成了一批新项目，打造了一批新亮点，城市品位形象得到新提升，百年工业城市越来越开放包容，越来越有生机活力，我们生活、奋斗和成长的家园，处处散发着现代化国际化的气息，彰显着和谐宜居美丽的品质。

这一年，我们以责任担当干成了一批大事难事，以改革创新破解了多年没有解决的历史遗留问题，以使命初心回应解决老百姓关心的身边事、具体事，全市各级干部群众对再创唐山发展新辉煌充满信心，780万英雄的唐山人民对唐山美好未来充满期待!

2018年目标任务

2018年是全面落实十九大精神的开局之年，是决胜全面建成小康社会、实施“十三五”规划承上启下的关键一年。前不久召开的市委十届四次全会，向全市发出了“牢记使命，解放思想，干在实处，走在前列，奋力谱写新时代唐山高质量发展新篇章”的号令动员，全面开启了新时代唐山改革发展的新征程。

走进新时代，踏上新征程，我们必须牢记历史使命，坚持以习近平新时代中国特色社会主义思想为统领，把党的十九大精神和总书记对唐山的重要指示熔铸在思想上、行动中，一步一个脚印扎实前进，加快实现“三个努力建成”，奋力谱写中华民族伟大复兴中国梦的唐山篇章。我们必须强化责任担当，把省委对唐山提出的“率先全面建成高质量小康社会和现代化沿海强市”目标要求，细化到每项任务、量化到每个节点，奋力推动唐山各项工作走在全省前列，在新时代全面建设经济强省美丽河北进程中展现首要首位、首责首任的唐山作为。我们必须时刻践行以人民为中心的发展思想，切实解决百姓关切的就业、就医、就学、养老、医疗、住房、环境等问题，在推动高质量发展中补齐民生短板，让全市人民在爱唐山、做贡献中感受共建共享的唐山温度，收获满满的获得感幸福感安全感。我们必须坚持实字当头、干字为先，紧紧抓住唐山加快新旧动能转换、实现由大到强战略性转变的历史机遇期和窗口期，以十项重点工作攻坚突破带动全局，把唐山的比较优势转化为发展胜势、汇聚成经济强势，以永不懈怠的精神状态和一往无前的奋斗姿态，干出新时代唐山发展的新天地，开创英雄城市的美好未来!

今年政府工作总体思路

以习近平新时代中国特色社会主义思想为指引，全面贯彻党的十九大精神和总书记“三个努力建成”重要指示，聚焦省委对唐山提出的“两个率先”目标要求，落实新发展理念，坚持稳中求进工作总基调，坚持高质量发展根本要求，以供给侧结构性改革为主线，统筹推进稳增长、促改革、调结构、治污染、惠民生、防风险，全力抓实建设生态唐山实现绿色发展、建设环渤海地区新型工业化基地、重大项目、“一港双城”、文化旅游产业融合、发展海洋经济和临港产业、优化营商环境、科技创新与新兴产业发展、人才队伍建设、保障和改善民生等十项重点工作，奋力谱写新时代唐山高质量发展新篇章。

2018年工作安排

今年经济社会发展的主要目标是：地区生产总值增长6.5%，一般公共预算收入增长7%，规模以上工业增加值增长5%以上，固定资产投资增长7%，实际利用外资增长5%，社会消费品零售总额增长10%;PM2.5平均浓度下降10.6%以上，每万元生产总值能耗和化学需氧量、二氧化硫、氨氮、氮氧化物减排完成省达任务;城镇和农村居民人均可支配收入均增长8%，城镇登记失业率控制在4%以内。这些指标的设定，既与省达计划和唐山“十三五”规划相衔接，又统筹了“稳”与“进”的平衡、“质”与“量”的协调、“破”与“立”的节奏，有利于经济运行稳控在合理区间，有利于调整优化经济结构、推动唐山高质量发展。

按照市委十届四次全会要求，围绕实现上述目标，重点抓好八方面工作：

1、聚焦建设现代化经济体系，加快推进唐山产业转型升级和结构优化

一是项目建设提效突破。聚焦“早”“快”“实”抓项目，完善以季度为单元的项目集中开工和观摩测评机制，精准解决供地、供电、供水、环评等问题，提高土地节约集约利用率和项目“亩均效益”，以优质新增量支撑高质量发展。加快推动京唐城际、唐曹铁路延伸至海港开发区。深化开发区管理体制、人事薪酬制度等改革，突出项目建设和招商引资主责主业，打造富有创新活力和竞争力的产业发展高地。二是新兴产业提速增量。保持战略定力，以久久为功的长跑思维推进新兴产业增速提高、占比提升，打造高质量发展新优势。三是传统产业提质增效。制定出台《加快建设环渤海地区新型工业化基地实施意见》，以“四新”促“四化”，推动传统产业向中高端跃升、向下游产业链延伸。四是现代服务业提档升级。以打造“唐山周末”品牌为目标导向，面向京津客源优化旅游资源配置，促进旅游业快速发展，带动城市转型，塑树唐山开放包容友好的城市形象。五是现代物流业提标创优。突出现代物流业在经济发展中的基础性、战略性作用，围绕打造一批多式联运工程、建设一批重点物流园区、引入一批大型物流企业、发展一批物流集散中心、整合一批物流信息平台，高起点制定产业规划，整合提升资源要素，构建“铁海港公空”立体化物流发展格局，努力打造立足京津冀、服务环渤海、对接东北亚的现代化国际物流中心。

2、围绕“破”“立”“降”深化供给侧结构性改革，提升经济发展质量效益

一是以化解过剩产能破除低端无效供给。严格落实“四个决不允许”要求，坚持完成化解任务、实施减量置换、推进退城搬迁三措并举，确保过剩产能只减不增、真去真退。二是以科技创新支撑新动能加速成长。围绕把科技创新变成实实在在的产业活动，抓实“京津孵化、唐山产业化”，年内引进京津等地科技成果转化项目60项以上，新增规模以上工业企业研发机构80家以上、高新技术企业100家、科技型中小企业1000家，全社会研发投入占GDP比重达到1.6%左右。三是以增强制度供给降低实体经济成本。用好国家产业转型升级示范区、小微企业创业创新基地城市示范等支持政策，打好用电、融资、物流、税费等降费减负“组合拳”，全年降低实体经济成本50亿元以上。

3、开掘释放大美海洋天然良港优势，打造沿海临港产业带

一是提升港口综合能级。统筹规划“一港三区”功能定位，探索建立资源整合、资产融合、资本聚合的协同开放体制，加快打造世界一流的国际化综合大港。二是打造曹妃甸增长极。突出曹妃甸沿海开发的龙头带动作用，全力推进金隅·曹妃甸协同发展示范产业园等项目建设，中冶瑞木新材料产业园、新澳肉牛屠宰加工等项目竣工投产，天坛家居、森工木业等50个木材加工贸易项目建成运营，年内曹妃甸区地区生产总值增长8%，规模以上工业增加值增长9%以上，产业投资增长20%以上。三是大力发展海洋经济。坚持生态开发、永续发展理念，集中开展海洋经济普查，高标准编制发展规划，全力培育发展海洋生物医药、海洋航运物流等产业，向海洋要空间要效益。四是加快临港产业聚集。优化提升临港工业、滨海旅游等产业布局，充分发挥曹妃甸、乐亭、海港、滦南滨海有港优势，加快发展精品钢铁、现代化工等临港产业，中铁十六局高端装备制造、安通国际多式联运智慧物流园等项目落地开工，境界高清洁燃料二期等项目加快建设。

4、优化“双城”空间布局，提升城市现代化国际化水平

一是以打造优质板块拓展内涵增长新空间。高水平建设城南经济开发区，高品质建设京唐智慧港，完善京唐城际机场站区域生活配套，高站位建设站西片区，高标准建设南湖CBD，高起点建设唐山冶金矿山机械厂区域。二是以增添魅力活力做强城市经济新支撑。推动文化旅游融合发展，提升运营唐山南站—古冶站铁路客运，促进要素流动和人员往来，谋划建立公共美术馆，完成唐山博物馆改陈提升，积极申办全国工业旅游创新会议，办好中国唐山评剧艺术节，以市场化理念提升唐山中国陶瓷博览会办会水平，高水平承办首届中国地震科普大会，全年举办专业展会70项，会展业直接收入增长20%以上。举办第三届中拉沙滩足球锦标赛等重大赛事活动10项以上，筹办国际信鸽大赛、世界雪日暨国际儿童滑雪节等特色赛事，年内体育产业增加值突破30亿元。三是以打通堵点制约推动承载能力新提升。翻修改造龙泽路、胜利路等城市道路，加快推进城中村搬迁，大力破解供电设施滞后城市发展的瓶颈制约，补齐会展场馆短板。四是以经营城市理念打造精明增长新模式。健全完善土地收储制度，合理调控供地节奏和区域，坚持“房子是用来住的、不是用来炒的”定位，优化城市资产市场化运作机制，深入推进城管执法体制改革。五是以唐山市主城区和曹妃甸滨海新城引领全域协调发展新格局。出台《推进“一港双城”建设实施意见》，7月底前完成城市总体规划修编，进一步明晰

主城区、曹妃甸、各县(市)空间布局和功能定位，实现错位融合发展。高标准修订曹妃甸城市总体规划和专项规划，加快实施新城城市运维中心、富力海时代会展中心等项目，谋划实施铁路编组站、游轮母港、曹妃甸通用机场建设，加快打造港产城融合的现代化滨海新城。培育壮大县域经济，促进产城教融合发展，加强财源建设，提升综合实力，力争涌现更多全国百强县。

5、拓展改革开放深度广度，激发高质量发展新动力

一方面，发挥重点领域改革牵引作用，打响唐山“四最”营商环境品牌。以刀口向内的决心和魄力，下大力减事项、减次数、减材料、减时间，大力推行“最多跑一次”改革和网上审批，打造唐山审批事项最少、收费标准最低、办事效率最快、服务水平最优的“四最”金字招牌。加强社会诚信体系建设，尊重、支持和爱护企业家，构建亲清新型政商关系。深入开展“双创双服”活动，最大限度便企利民。推进财政事权与支出责任划分改革，完成新城建集团市场化转型。深化行政管理体制和事业单位改革，加强和规范机构编制管理，优化配置机构编制资源。深化公立医院综合改革，加快推进分级诊疗、医联体建设，让群众在家门口享受优质医疗服务。

另一方面，坚持“引进来”“走出去”并重，全方位扩大对外开放。持续开展承接京津产业转移招商行动，芦台、汉沽聚焦现代家居等优势产业，打造特色产业集群，精准开展以商招商、驻点招商和“凤还巢”工程，充分发挥异地唐山商会带动作用，深入开展政府对话、企业合作、民间交流等活动，加快建设华通线缆哈萨克斯坦经济园区等重点项目，新缔结2个国际友好城市，拓展唐山海外“朋友圈”。

6、抓实乡村振兴战略，做好新时代三农工作

一是推进农业供给侧结构性改革。坚持质量兴农、绿色兴农，创建国家农产品质量安全市，“三品一标”和品牌农产品占比达到55%。严格落实粮食安全责任制，加强流通监管，确保粮食安全。实施省级“粮改饲”试点市项目，大力发展渔业生态健康养殖和休闲渔业。推进农村一二三产业融合发展，提升现代农业园区建设水平。二是加快建设美丽宜居乡村。以垃圾处理、污水治理、厕所改造、村容村貌整治为重点，实施农村人居环境整治三年行动计划，不断提升农村环境质量。加快实施农村饮水安全工程，新建改建农村公路400公里。三是深化农村综合改革。统筹推进农村承包地“三权分置”、农村集体产权制度改革、农垦改革、供销社综合改革等工作，释放农村发展新活力。

7、坚持生态唐山、绿色发展，打好污染防治攻坚战

一是打赢蓝天保卫战。精准落实“清、准、高、快、狠”五字要求，强力实施“退十计划”，以壮士断腕的决心，下足绣花的功夫，抓好每一天、每一项环保治理，年内PM2.5平均浓度控制在59微克/立方米左右，空气质量综合指数控制在7.0左右，让老百姓享受更多的幸福蓝天。健全完善市县乡村网格化体系，以建筑工地“六个百分之百”为首战，发现一起查处一起，确保令行禁止、铁腕治污。精准实施差异化“双错峰”和应急响应，依据企业环保水平实施阶梯管控、动态管理。扎实开展工业污染源深度治理，强化企业关键工序减排。积极推进唐山港疏港运输“公转铁”，规划建设钢铁企业铁路专用线，加快构建海铁联运新体系，充分释放疏港运力潜能，有效破解严重依赖汽运造成的污染和超载问题。二是统筹推进山水林田湖海生态修复。严守生态红线，全面落实“水十条”“土十条”。深入实施河长制、湖长制，开展清岸、还绿、纳网、提标、增容“五大工程”，确保国控省控河流断面稳定达标、清水长流。强化陡河水库、上关水库等饮用水源地保护，全面消除城市黑臭水体，工业园区污水集中处理实现全覆盖。加强港口污染综合治理，提高岸基供电使用率，海洋运输船舶在近海海域一律使用轻质油。强力推进土壤污染防治，开展国土绿化三年行动，积极创建国家森林城市。三是最大限度节能减排降耗。严格能源消费总量和强度双控制度，开展重点用能单位“百千万”行动，完成省达削减煤炭总量任务。推进冬季清洁取暖，全面取缔禁燃区内散煤经销网点，实现城中村取暖清洁化。抓好20个资源综合利用和循环化改造项目，节能环保产业增加值增长12%以上。倡导简约适度、绿色低碳生活方式，创建节约型机关和绿色家庭、绿色学校、绿色社区。

8、顺应人民美好生活需要，增强群众获得感幸福感安全感

一是让群众享受更加普惠均等的公共服务。持续加大民生投入，办好实事工程，确保幼有所育、学有所教、劳有所得、病有所医、老有所养、住有所居、弱有所扶落到实处。二是让群众享受更加丰富多彩的美好生活。弘扬社会主义核心价值观，巩固提升全国文明城创建成果，注重家庭、家教、家风建设。加快落实总书记见证签约的唐山市与乌拉圭政府足球计划协议，推动乌拉圭、北京、唐山三方共建国际足球学校和人才培养交流，打造中乌足球领域双边合作示范项目。三是让群众享受更加和谐顺心的社会环境。守住安全稳定底线，统筹推进安全生产、交通安全、消防安全、食药品安全等工作，有效防控风险隐患，坚决杜绝重特大安全事故发生。

努力建设人民满意政府

以正确航向引领思想解放，提升推动高质量发展的能力水平。进一步学懂弄通做实十九大精神，坚决做到信念过硬、政治过硬、责任过硬、能力过硬、作风过硬，真正把“四个意识”践行到岗位上、行动中。深入开展“三个看一看”解放思想大讨论活动，引导政府系统转变观念、开拓视野，以新理念、新思维加快推动唐山转型发展、高质量发展。

以事争一流标准改革创新，在应对困难挑战中闯出新路走在前列。坚持高点“选标定位”，跳出唐山看唐山，站位河北争一流，放眼全国敢争比，做到善作善成、久久为功。突出目标需求和问题导向，聚焦影响发展的关键点、制约点，化解老问题，解决新矛盾。强化改革创新意识，着力在发挥市场机制、推进产业转型、发展开放型经济等方面先行先试，塑造更多依靠创新驱动、发挥唐山独特优势的引领型发展。

以事不隔夜状态狠抓落实，确保各项工作抓实干成。按照干什么、谁来干、怎么干、什么时间干成，对重项工作逐项列出时间表、路线图，落实专人专责，全方位跟踪、全过程调度，确保定下的事干一件、成一件。大力推行一线工作法，特别是对重大项目建设、大气污染治理、“四最”环境品牌等重点工作，政府系统各级干部既挂帅又出征，一抓到底，实干实效。

以锲而不舍韧劲正风肃纪，营造清正清廉清明的政务环境。扎实开展“不忘初心、牢记使命”主题教育，驰而不息整治“四风”，把“三严三实”贯穿于工作生活全过程。坚持依法行政、从严治政，自觉接受人大依法监督、政协民主监督和社会舆论监督。压实全面从严治党责任，加强党风廉政建设，强化行政监察和审计监督，“底线、红线”一起守，“不为、乱为”一起治，树立新时代锐意进取、廉洁高效的政府形象。
